# Supplementary material for: ADAMTS13 in Bothrops lanceolatus snakebite envenoming: Crude venom-induced reduction of in vitro enzymatic activity and clinical correlation in snakebite patients
Source: PLoS Negl Trop Dis. 2025 Oct 29;19(10):e0013678. doi: 10.1371/journal.pntd.0013678 (PMC12591401; doi:10.1371/journal.pntd.0013678)
Supplement: S1 Table — (DOCX) [file pntd.0013678.s001.docx]

**Supplemental Table S1**: Main biological parameters of the 46 patients according to age >65-year-old.

|  | **All patients** | **Age < 65 years** | **Age > 65 years** | **P** |
| --- | --- | --- | --- | --- |
| **Biological analysis** |  |  |  |  |
| Hemoglobin (g/dL), IQR | 15.1 (14.1-16.3) | 15.2 (14.1-16.9) | 14.9 (13.1-16.1) | 0.834 |
| Leukocyte count (10^3^/µL), IQR | 7.7 (5.9-9.7) | 8.4 (6.4-10.7) | 6.6 (5.4-8.5) | 0.123 |
| Platelet count (Giga/L), IQR | 230 (199- 275) | 240 (221-292) | 172 (159-234) | 0.667 |
| Partial thromboplastin time, PTT(%), IRQ | 98 (93-107) | 97 (92-107) | 101 (94-115) | 0.456 |
| Prothrombin time, PT (%), IQR | 94 (84-107) | 97 (92-111) | 94 (88-109) | 0.876 |
| Fibrinogen (g/L), IQR | 3.2 (2.9-3.8) | 3.1 (2.8-3.5) | 3.2 (2.9-3.6) | 0.666 |
| C-reactive protein (mg/dL), IQR | 2.5 (0.6-7.2) | 2.8 (1.0-6.0) | 1.9 (1.0-7.2) | 0.213 |
| CPK (IU/L), IQR | 229 (146-336) | 223 (148-322) | 222 (149-345) | 0.678 |
| ADAMTS13 activity (%), IQR | 92.5 (77.5-116.8) | 94 (82-123) | 78 (77-134) | 0.121 |

Results are reported as median and inter-quartile range (IQR). Abbreviation: ADAMTS13, a disintegrin-like and metalloproteinase with a thrombospondin type 1 motif, member 13; CPK, creatine phosphokinase.
