# Supplementary material for: ADAMTS13 in Bothrops lanceolatus snakebite envenoming: Crude venom-induced reduction of in vitro enzymatic activity and clinical correlation in snakebite patients
Source: PLoS Negl Trop Dis. 2025 Oct 29;19(10):e0013678. doi: 10.1371/journal.pntd.0013678 (PMC12591401; doi:10.1371/journal.pntd.0013678)
Supplement: Attachment — Submitted filename: PNTD-D-25-01531_reviewer.pdf [file pntd.0013678.s004.pdf]

PLOS Neglected Tropical Diseases

ADAMTS-13 activity is reduced by Bothrops lanceolatus snake venom: in vitro experiments and clinical correlation

--Manuscript Draft--

**Manuscript Number:**	PNTD-D-25-01531
**Full Title:**	ADAMTS-13 activity is reduced by Bothrops lanceolatus snake venom: in vitro experiments and clinical correlation
**Short Title:**	ADAMTS-13 activity and Bothrops lanceolatus envenoming
**Article Type:**	Research Article
**Keywords:**	snakebite, envenoming, Bothrops lanceolatus, ADAMTS13, thrombosis, inflammation, prognosis
**Abstract:**	Background<br>Envenoming by Bothrops lanceolatus, a viperid species endemic to Martinique an island in the Lesser Antilles, induces a unique clinical manifestation, i.e., thrombosis. Pathophysiological signaling leading to thrombotic events is largely debated. Among others, proposed mechanisms include increased expression of multimerized forms of von Willebrand factor (VWF). Prothrombotic effects of VWF are negatively regulated by ADAMTS13, a metalloprotease which cleaves VWF multimers. Whether ADAMTS13 activity is reduced in B. lanceolatus envenoming has not been previously investigated.<br>Methodology/Principal Findings<br>ADAMTS13 activity was evaluated via chromogenic assay in experimental and clinical studies. Human plasma with known ADAMTS13 activities (0.70 IU/mL) wa incubated with increasing doses of B. lanceolatus venom. Incubation with B. lanceolatus venom (concentrations 10-1000 ng/mL) induced a dose-dependent reduction of ADAMTS-13 activity, while B. atrox venom had opposite effects. In a large series of 46 patients bitten by B. lanceolatus, ADAMTS-13 activity was determined at admission before antivenom therapy. The median plasmatic ADAMTS13 activity was 94% (IQR: 78–122%), while ADAMTS13 activity less than 75% was observed in ten (22%) patients. Compared with patients with normal ADAMTS13 activity (n=36), patients with moderately low ADAMTS13 activity (n=10) were older and displayed a lower platelet count, a more intense myonecrosis along with higher fibrinogen and C-reactive protein levels. Multivariate linear regression retained only grade severity of envenoming as independent predictor of increase length of hospital stay, while reduced ADAMTS13 activity and increased C-reactive protein levels bordered on statistical significance.<br>Conclusions/Significance<br>For the first time, our study provided evidence suggesting that ADAMTS-13 activity is reduced in experimental B. lanceolatus venom exposure and patients with B. lanceolatus envenoming. Thanks to its role on the VWF regulation, it is suggested that reduced ADAMTS-13 activity can increase the risk of thrombosis in B. lanceolatus envenoming by favoring the circulation of prothrombotic VWF multimers.
**Additional Information:**	
**Question**	**Response**
**Government Employee**<br><br>Are you or any of the contributing authors an employee of the United States government?<br><br>Manuscripts authored by one or more US Government employees are not copyrighted, but are licensed under a CC0 Public Domain Dedication, which allows	No - No authors are employees of the U.S. government.

Powered by Editorial Manager® and ProduXion Manager® from Aries Systems Corporation

unlimited distribution and reuse of the article for any lawful purpose. This is a legal requirement for US Government employees.<br><br>This will be typeset if the manuscript is accepted for publication.	
**Financial Disclosure**<br><br>Enter a financial disclosure statement that describes the sources of funding for the work included in this submission. Review the submission guidelines for detailed requirements. View published research articles from *PLOS Neglected Tropical Diseases* for specific examples.<br><br>This statement is required for submission and **will appear in the published article** if the submission is accepted. Please make sure it is accurate.<br><br>**Funded studies**<br>Enter a statement with the following details:<br>• Initials of the authors who received each award<br>• Grant numbers awarded to each author<br>• The full name of each funder<br>• URL of each funder website<br>• Did the sponsors or funders play any role in the study design, data collection and analysis, decision to publish, or preparation of the manuscript?<br><br>Did you receive funding for this work?	The author(s) received no specific funding for this work.
**Competing Interests**<br><br>On behalf of all authors, disclose any competing interests that could be perceived to bias this work.<br><br>This statement will be typeset if the manuscript is accepted for publication.	Authors have no conflict of interest

Review the instructions link below and PLOS NTDs' competing interests policy to determine what information must be disclosed at submission.	
**Data Availability**<br><br>Provide a **Data Availability Statement** in the box below. This statement should detail where the data used in this submission can be accessed. This statement will be typeset if the manuscript is accepted for publication.<br><br>Before publication, authors are required to make all data underlying their findings fully available, without restriction. Review our PLOS Data Policy page for detailed information on this policy. Instructions for writing your Data Availability statement can be accessed via the Instructions link below.	data are included in Supporting Information files of the submitted manuscript

Manuscript

Click here to access/download;Manuscript;_PlosNTD_ADAMTS-

ADAMTS-13 activity is reduced by *Bothrops lanceolatus* snake venom: *in vitro* experiments and clinical correlation

Short title: ADAMTS-13 activity and *Bothrops lanceolatus* envenoming

Jonathan FLORENTIN * [1,2],

Caroline RAPON * [1],

Olivier PIERRE-LOUIS [1],

Fatima RADOUANI [1,3],

Prisca JALTA [1,3],

Pascal FUSEAU [4]

Hatem KALLEL [5]

Remi NEVIERE [1]

Dabor RESIERE [1,2],

[1] Cardiovascular Research Team (UR5_3 PC2E), University of the French West Indies (Université des Antilles), Fort de France, France.

[2.] Department of Toxicology and Critical Care Medicine, University Hospital of Martinique (CHU Martinique), Fort-de-France, France

[3] Department of Research, University Hospital of Martinique (CHU Martinique), 97261 Fort-de-France, France

[4] Department of Biology – Hematology, University Hospital of Martinique (CHU Martinique), Fort-de-France, France.

[5] Intensive Care Unit, Cayenne General Hospital, and Tropical Biome and Immunopathology CNRS UMR-9017, Inserm U 1019, Université de Guyane, Cayenne, French Guiana.

(*) Equally contributed

Corresponding author: Pr. Remi NEVIERE, Department of Clinical Physiology, CHU Martinique (University Hospital of Martinique), Route de Chateauboeuf, CS 90632, 97261 Fort de France, FRANCE – Tel: (+596) 596 55 98 73, email: remi.neviere@chu-martinique.fr

ABSTRACT

Background

Envenoming by *Bothrops lanceolatus*, a viperid species endemic to Martinique an island in the Lesser Antilles, induces a unique clinical manifestation, i.e., thrombosis. Pathophysiological signaling leading to thrombotic events remains poorly understood. Among others, proposed mechanisms include increased expression of multimerized forms of von Willebrand factor (VWF). Prothrombotic effects of VWF are regulated by ADAMTS13, a metalloprotease which cleaves VWF multimers. Whether ADAMTS13 activity is reduced in *B. lanceolatus* envenoming has not been previously investigated.

Methodology/Principal Findings

ADAMTS13 activity was evaluated via chromogenic assay in experimental and clinical studies. Human plasma with known ADAMTS13 activities (0.70 IU/mL) was incubated with increasing doses of *B. lanceolatus* and *B. atrox* venoms. Incubation with *B. lanceolatus* venom (concentrations 10-1000 ng/mL) induced a dose-dependent reduction of ADAMTS-13 activity, while *B. atrox* venom had opposite effects. In our series of 46 patients bitten by *B. lanceolatus* snake, ADAMTS-13 activity was determined at admission before antivenom therapy. In these patients, the median plasmatic ADAMTS13 activity was 94% (IQR: 78–122%). Ten patients (22%) displayed ADAMTS13 activity less than 75%. Compared with patients with normal ADAMTS13 activity (n=36), those with moderately low ADAMTS13 activity (n=10) were older, had a lower platelet count, and displayed increased concentrations of creatine kinase, fibrinogen and C-reactive protein. Multivariate linear regression retained only grade severity of envenoming as independent predictor of increase length of hospital stay, while reduced ADAMTS13 activity and increased C-reactive protein levels bordered on statistical significance.

Conclusions/Significance

For the first time, our study provided evidence suggesting that ADAMTS-13 activity is reduced in experimental *B. lanceolatus* venom exposure and patients with *B. lanceolatus* envenoming. Thanks to its role on the VWF regulation, it is suggested that reduced ADAMTS-13 activity can increase the risk of thrombosis in *B. lanceolatus* envenoming by favoring the circulation of prothrombotic VWF multimers.

Key words: snakebite, envenoming, *Bothrops lanceolatus*, ADAMTS13, thrombosis, inflammation, prognosis

Author Summary

The clinical manifestations of envenoming by *Bothrops* sp. typically include local tissue injury and disturbances of hemostasis characterized by consumption coagulopathy leading to incoagulable blood. In contrast, *B. lanceolatus* envenoming can elicit thrombotic events, such as ischemic stroke and myocardial infarction. Proposed mechanisms include the combination of procoagulant and systemic inflammatory processes leading to thrombotic state. The factor von Willebrand (VWF) is an active biomolecule critical for coagulation and thrombosis processes. The amount of multimerized forms of VWF increases the risk of thrombosis. VWF is regulated by ADAMTS13, an enzyme that cleaves VWF multimers and reduced their prothrombotic effects. Whether ADAMTS13 activity is reduced in *B. lanceolatus* envenoming has not been previously investigated, while only few case reports have previously evaluated the levels of ADAMTS-13 activity in human envenoming. For the first time in this study, we study provided evidence suggesting that ADAMTS-13 activity is reduced in experimental *B. lanceolatus* venom exposure and in human *B. lanceolatus* envenoming. Our study results further suggest that, together with high grade severity score of envenoming and systemic inflammation, moderately reduced levels of ADAMTS-13 activity are associated with increased length of hospital stays.

INTRODUCTION

The clinical manifestations of envenoming by *Bothrops* sp. include local effects, such as pain, edema, and necrosis, and systemic complications, such as disturbances of hemostasis characterized by consumption coagulopathy and defibrinogenation, and, in some cases, acute kidney failure and circulatory shock [1-3]. In contrast to the typical hemostatic alterations induced by *Bothrops* sp. venoms, envenoming by *B. lanceolatus* can elicit diffuse thrombotic microangiopathy and severe thrombotic events within 48h of the bite [4-6].

B. lanceolatus is a viperid species endemic to Martinique, an island in the Lesser Antilles of the West Indies in the south of the Caribbean Sea. Before the era of immunotherapy, *B. lanceolatus* envenoming was associated with systemic arterial thrombotic complications leading to cerebral, myocardial or pulmonary infarctions in 25-30% of cases and was fatal in about 10% of cases [7-11]. Since the mid 1990s, the use of the monospecific antivenom Bothrofav® (Micropharm, UK), has reduced mortality to zero and prevented most of the thrombosis complications [7-11]. Despite excellent efficiency of Bothrofav®, the exact pathophysiological mechanisms leading to *B. lanceolatus* venom-induced thrombotic complications remain poorly understood. In absence of overt proaggregating or procoagulant activities, proposed mechanisms of *B. lanceolatus* venom-induced thrombosis include increased expression of tissue factor, complement system activation, endothelial dysfunction and immune inflammatory systemic response [10,12-14].

Biological features consistent with thrombotic microangiopathy has been described after snakebite for many years [15,16]. In viperid envenoming, thrombotic microangiopathy has been previously described in some *Bothrops* sp. envenoming, including by *B. jararaca*, *B. venezuelensis* and *B. erythromelas* [17-19]. In these cases, patients presented on admission a typical local bothropic syndrome with no signs of systemic bleeding, whereas thrombotic microangiopathy can developed within the few days after snakebite [15-19]. Of note, thrombotic microangiopathy is the overarching term used to describe any disease process characterized by thrombocytopenia and microangiopathic hemolytic anemia with or without other clinical or laboratory features (thrombocytopenia, hemolytic anemia with presence of schistocytes in blood smears, decreased haptoglobin level, increased lactate dehydrogenase level and acute kidney injury) [20,21]. Primary thrombotic microangiopathies include hemolytic uremic syndrome (HUS) and thrombotic thrombocytopenic purpura (TTP). Typical TTP is broadly defined as a thrombotic microangiopathy occurring in the context of severe ADAMTS13 deficiency (<10%), while ADAMTS13 is usually not decreased in HUS [20-22]. Secondary forms of thrombotic microangiopathies, which occur in the context of pregnancy, autoimmune disease, malignancy, bone marrow transplantation or use of certain medications, are associated with mildly reduced or even normal ADAMTS13 activity [21-23].

ADAMTS13 is a disintegrin-like and metalloproteinase with a thrombospondin type 1 motif, member 13, which regulates hemostasis and thrombosis processes related to von Willebrand factor (VWF) pathway. VWF activity is regulated by ADAMTS13, an enzyme that cleaves VWF multimers and reduced their prothrombotic effects [23]. Increasing evidence suggest that even moderate decreases of ADAMTS-13 activity can also predispose to ischemic cardiovascular diseases, particularly myocardial infarction and ischemic stroke [24,25]. Previous experimental reports in *Bothrops* sp. envenoming have described mild reduction of ADAMTS13 activity [26,27]. In *B. jararaca* envenoming, reduction of ADAMTS13 was associated with reduced VWF levels and cleavage of high molecular weight VWF multimers [26]. *B. jararaca* venom was also shown to downregulate mRNA synthesis of hepatic *Adamts13* [28]. In human envenoming, only few case reports have previously evaluated the levels of ADAMTS-13 activity, which were normal when tested [17,18,29-31].

Whether reduced ADAMTS-13 activity would play a role in the endothelial dysfunction and risk of thrombosis associated with *B. lanceolatus* envenoming have not been studied. We hypothesized that moderately reduced levels of ADAMTS13 activity may be involved in the pathophysiological mechanisms of the thrombotic complications associated with *B. lanceolatus* envenoming. Main objectives of our study were to test whether plasmatic ADAMTS-13 activity would be reduced by *in vitro* exposure to *B. lanceolatus* venom (in contrast with *B. atrox* venom) and in humans bitten by *B. lanceolatus* snake.

PATIENTS AND METHODS

Venom and antivenom

The crude venoms were obtained from adult wild-caught *B. lanceolatus* (n=12) and *B. atrox* specimens (n=5), captured in Martinique and French Guyana, respectively. Venom samples were pooled, lyophilized (Freezone, Labconco, Kansas City, MO, USA) and stored at -80°C until use (stock solution 10 mg/mL). In *in vitro* experiments, *B. lanceolatus* and *B. atrox* venoms were used at final concentrations ranging from 10 to 1000 ng/mL.

Bothrofav® (Micropharm, UK), a preparation containing F(ab')2 fragments that have the property of neutralizing *B. lanceolatus* venom, was administered in clinical *B. lanceolatus* envenoming. These equine F(ab')2 fragments ligate venom antigens present in circulating blood to form inactive F(ab')2-antigen complexes, in turn reducing the amount of free venom in circulation. Bothrofav® was used (batch J8216; protein concentration of 20.7±0.05 g/dL) at the final concentrations of 0.21 mg/mL.

Assay of ADAMTS13 activity

Levels of ADAMTS13 activity were measured via a chromogenic ADAMTS13 activity ELISA kit (Technozym®, Cryopep, France). The principle of the assay is related to the incubation of plasma with

a fragment of VWF (GST-vWF73 substrate). The specific cleavage of this fragment by ADAMTS-13 is then detected using a labeled antibody targeting the cleaved VWF fragment. The intensity of the staining is directly proportional to the amount of cleaved substrate and, therefore, to the ADAMTS-13 activity in the plasma sample. According to manufacturer's instructions, incubation times for the GST-vWF73 substrate and study sample were 60 min and 30 min, respectively. Incubation times for the conjugated antibody and chromogenic substrate were 60 min and 30 min, respectively. The reaction was stopped and optical density was measured at 450 nm. Calibrators from 0 to 1 IU/mL (Cal Set 4-5450761, Technozym®) and controls (Control Set 4-5450763, Technozym®) of 0.70 IU/mL concentration were used for the determination of ADAMTS-13 activity. Controls and calibrators have been calibrated against the 1st WHO international standard (NIBSC code 12/252) in IU/mL. Activity of ADAMTS-13 was expressed as percent of controls.

Patients

Patients with the diagnosis of *B. lanceolatus* envenoming presenting at the University Hospital of Martinique from January 2019 to Janvier 2023 were retrospectively. Information regarding the possible use of the clinical records for research was provided to patients at time of hospitalization and consent was written in the medical file of the patient. All patients were managed in accordance with the amended Declaration of Helsinki (http://www.wma.net/ en/30publications/10policies/b3/). The study was approved by the CHU Martinique hospital's institutional review board (IRB #01072019).

Diagnosis and antivenom therapy of *B. lanceolatus* snakebite envenoming

When seen, the culprit snake was identified based on the patient's description, photographs, or the physical examination of the captured snake. Snakebite was confirmed by the observation of two puncture wounds resulting from snake fang marks. Patient health data were collected using the Emergency DX Care software (Medasys, Dedalus, France) on hospital presentation. Data recording and patient management was performed with the help of dedicated senior toxicologist physicians of the snakebite team at the CHU Martinique. Clinical data, including age and gender of patients, date and time of the bite, anatomical site of the bite, grade of severity of envenoming, clinical manifestations and routine biological tests at admission, date and time of hospital admission, and antivenom administration were collected from the DX Care medical record of the patients. For ADAMTS13 activity assay, peripheral whole blood was collected with sodium citrate at admission All samples were stored at −80 °C, thawed at 37 °C immediately before testing, and were not refrozen. The severity of envenoming of each patient was measured according to a previously defined clinical severity scale. Development of envenoming, occurrence of adverse events, duration of hospital stays, and outcome were monitored for each patient. We excluded patients hospitalized for snakebite envenoming lasting more than 48 hours after the bite and those who did not present clinical or biological signs of envenoming. Envenomed

patients were treated by intravenous infusion of antivenom Bothrofav® at dose adjusted to the severity grade of the case.

Statistical analysis

Quantitative data were presented as mean ± standard deviation (SD) or median IQR. The Shapiro-Wilk test was used to test for normal distribution of quantitative data. Median, interquartile range, and minimum–maximum ranges are described for non-normally distributed variables. Categorical data are presented as absolute values with percentages. The following tests were used for group comparisons: Student's t-test, analysis of variance ANOVA, chi-square test, and Fisher's exact test. When a significant difference was found, we identified specific differences between groups with a sequentially rejective Bonferroni procedure. Level of statistical significance was set at $p < 0.05$. In this exploratory work, univariate and multivariate linear regression models were implemented to assess the independent effect of potential predictors of length of hospital stay in the study population. All statistical analyses were conducted using IBM SPSS® Statistics version 27 for Windows software. Figures were processed using Prism 6 for Windows software (Graphpad, Boston MA, USA).

RESULTS

In vitro effects of B. lanceolatus venom on plasmatic ADAMTS13 activity

Venom incubation with plasma samples displaying a fixed ADAMTS13 activity induced optical density changes of the plasma staining, which is directly proportional to the amount of cleaved chromogenic substrate and, therefore, the ADAMTS-13 activity in the sample (Figure 1). Incubation of plasma displaying a known ADAMTS13 activity (0.70 IU/mL) with *B. lanceolatus* venom induced a dose dependent (1-1000 ng/mL) reduction of ADAMTS13 activity (Figure 1A). In contrast, incubation with *B. atrox* venom at concentrations ranging from 10 to 100 ng/mL increased ADAMTS13 activity, while a concentration of 1000 ng/mL had not effects on ADAMTS13 activity.

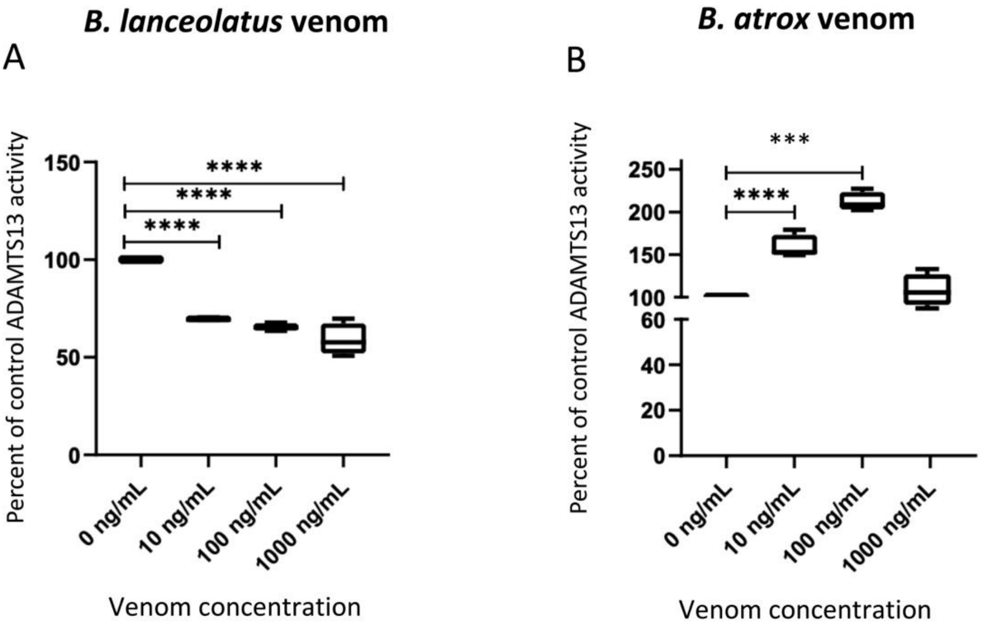

Figure 1: Representative ADAMTS13 activity changes following *B. lanceolatus* (panel A) and *B. atrox* (panel B) venom incubation with plasma samples displaying a fixed ADAMTS13 activity (0.70 IU/mL) in human plasma. Experiments were performed with *B. lanceolatus* and *B. atrox* venom concentrations ranging from 10 to 1000 ng/mL. Results are displayed as box plot with median, min to max data points. N=5-6 experiments, *** indicates p<0.01, **** indicates p<0.001.

Main characteristics and ADAMTS13 activity in human *B. lanceolatus* envenoming

From January 2019 to Janvier 2023, a total of 116 patients suffering envenoming by *B. lanceolatus* were included in the study. Patient health data was carefully collected using the Emergency DX Care software (Medasys, Dedalus, France) on hospital presentation. Patient's registry, critical information such as clinical and biological presentation, envenoming grade severity, timing of antivenom administration, antivenom protocol, thrombotic complications and follow-up were fully available (Table 1). Among the 116 envenomed patients, a total of 46 patients (39.6%) had have ADAMTS13 activity determination at admission before antivenom therapy. There were no differences in terms of clinical conditions, main snakebite characteristics and grade severity between patients who had or not ADAMTS13 activity determination (Table 1).

<u>Table 1</u>: Baseline characteristics of patients bitten by *B. lanceolatus*

	**All patients**	**Patients with ADAMTS13**	**Patients without ADAMTS13**	**P**
Number of cases	116	46	70	

Age (years), IQR	50 (36-60)	49 (36-60)	50 (39-60)	0.919
Gender (male), n (%)	75 (64.7%)	32 (69.6%)	43 (61.4%)	0.2434
Cardiovascular risk factors, n (%)	35 (30%)	17 (37%)	18 (26%)	0.139
**Snakebite presentation**				
Agriculture, n (%)	38 (33%)	14 (30%)	24 (34%)	0.691
Trekking, n (%)	13 (11%)	7 (10%)	6 (13%)	0.412
Snake identified, n (%)	28 (24%)	13 (28%)	15 (21%)	0.266
Upper limb site of bite, n (%)	25 (22%)	8 (17%)	17 (24%)	0.259
Lower limb site of bite, n (%)	91 (78%)	53 (76%)	38 (83%)	0.259
Delay for antivenom (hrs:min), IQR	2:49 (2:00-5:05)	2:17 (1:51-3:30)	3:00 (2:10-5:40)	0.225
**Grade severity of envenoming**				0.094
Grade 1, n (%)	5 (4.3%)	0 (0%)	5 (7.1%)	-
Grade 2, n (%)	92 (79.3%)	36 (78.3%)	56 (80.0%)	-
Grade 3, n (%)	13 (11.2%)	7 (15.2%)	6 (8.6%)	-
Grade 4, n (%)	6 (5.2%)	3 (6.5%)	3 (4.3%)	-
**Clinical features / complications**				
Systolic arterial pressure, IQR	143(123-156)	138 (129-151)	146 (122-160)	0.575
Heart rate (bpm), IQR	80 (69-90)	81 (69-91)	80 (69-90)	0.989
SpO_2 (%), IQR	98 (97-100)	98 (97-100)	98 (98-100)	0.515
Temperature (°C), IQR	36.7 (36.5-37.0)	36.8 (36.6-37.1)	36.4 (36.6-36.9)	0.141
Stroke, n (%)	2 (2%)	0 (0%)	2 (2.9%)	0.362
**Biological analysis**				
Hemoglobin (g/dL), IQR	15.3 (14.3-16.5)	15.1 (14.1-16.3)	15.4 (14.6-16.8)	0.767
Leukocyte count (10^3/µL), IQR	7.6 (5.8-9.8)	7.7 (5.9-9.7)	7.5 (5.8-9.9)	0.287
Platelet count (Giga/L), IQR	240 (204-272)	230 (199-275)	243 (205-266)	0.669
Thrombocytopenia, n (%)	5 (4.3%)	2 (4.3%)	3 (4.3%)	0.990
Partial thromboplastin time, PTT (%), IQR	100 (93-107	98 (93-107)	101 (93-109)	0.759
Prothrombin time, PT (%), IQR	95 (87-106)	94 (84-107)	95 (89-106)	0.872
Fibrinogen (g/L), IQR	3.2 (2.9-3.9)	3.2 (2.9-3.8)	3.3 (2.8-4.0)	0.640
C-reactive protein (mg/dL), IQR	2.6 (0.7-7.9)	2.5 (0.6-7.2)	3.0 (0.7-9.8)	0.129
CPK (IU/L), IQR	222 (122-377)	229 (146-336)	211 (116-465)	0.647

ADAMTS13 activity (%), IQR		92.5 (77.5-116.8)		na
ADAMTS13 activity <75%, n (%)		10 (22%)		na
**Treatment**				
Bothrofav® administration, n (%)	110 (95%)	45 (98%)	65 (93%)	0.232
Antibiotics, n (%)	25 (22%)	12 (26%)	13 (19%)	0.231
**Outcome**				
Ambulatory patients, n (%)	57 (49%)	22 (48%)	35 (50%)	0.484
Length of hospital stay (day), mean±SD	2.0±1.9	2.0±1.8	2.1±1.9	0.969
Deceased patients, n (%)	0 (0%)	0 (0%)	0 (0%)	na

Results are reported as median and inter-quartile range (IQR) or as absolutes number of cases and percentages. Abbreviation: ADAMTS13, a disintegrin-like and metalloproteinase with a thrombospondin type 1 motif, member 13; CPK, creatine phosphokinase; MRI, magnetic resonance imaging; na, non-applicable. Thrombocytopenia was defined as a platelet count of less than 150 Giga/L.

Main characteristics of the 46 patients with available ADAMTS13 activity determination are displayed Table 2. The median age of these 46 patients was 49 years (IQR: 36–60). The median time from the snakebite to hospital admission was 2:17 hours (IQR: 1:51–3:30). Most participants were male (70%). The envenomed patients were agricultural workers in 30% of cases. Moderate envenoming was the most common presentation (78%). Two patients developed ischemic cerebral stroke. The median plasmatic ADAMTS13 activity was 94% (IQR: 78–122%), while an ADAMTS13 activity less than 75% was observed in ten (22%) patients. This group of 10 patients were older and displayed a lower platelet count (which remain normal), a more intense myonecrosis along with higher fibrinogen and C-reactive protein levels (Table 2) compared with patients with normal ADAMTS13 activity.

Table 2: Baseline characteristics of patients bitten by *B. lanceolatus* with ADAMTS13 activity determination

	All patients	ADAMTS13 > 75%	ADAMTS13 <75%	P
Number of cases	46	36	10	
Age (years), IQR	49 (36-60)	47 (34-59)	58 (45-73)	0.025
Gender (male), n (%)	32 (70%)	23 (64%)	9 (90%)	0.143
Cardiovascular risk factors, n (%)	17 (37%)	13 (36%)	4 (40%)	0.550
**Snakebite presentation**				

Agriculture, n (%)	14 (30%)	12 (33%)	2 (20%)	0.347
Trekking, n (%)	7 (10%)	6 (17%)	1 (10%)	0.425
Snake identified, n (%)	13 (28%)	9 (25%)	4 (4(0%)	0.289
Upper limb site of bite, n (%)	8 (17%)	5 (14%)	3 (30%)	0.344
Lower limb site of bite, n (%)	38 (83%)	31 (82%)	7 (70%)	0.344
Delay for antivenom (hrs:min), IQR	2:17 (1:51-3:30)	2:17 (1:54-3:57)	2:00 (1:10-3.14)	0.301
**Grade severity of envenoming**				0.208
Grade 1, n (%)	0 (0%)	0 (0%)	0 (0%)	
Grade 2, n (%)	36 (78.3%)	29 (80.6%)	7 (70.0%)	
Grade 3, n (%)	7 (15.2%)	4 (11.1%)	3 (30.0%)	
Grade 4, n (%)	3 (6.5%)	3 (8.3%)	0 (0%)	
**Clinical features / complications**				
Systolic arterial pressure, IQR	138 (129-151)	138 (125-155)	143 (129-158)	0.581
Heart rate (bpm), IQR	81 (69-91)	81 (69-94)	70 (62-83)	0.078
SpO_2 (%), IQR	98 (97-100)	98 (97-100)	97 (96-100)	0.160
Temperature (°C), IQR	36.8 (36.6-37.1)	36.8 (36.7-37.1)	36.6 (36.6-37.1)	0.393
Stroke, n (%)	0 (0%)	0 (0%)	0 (0%)	na
Peripheral thrombosis, n (%)	1 (2.2%)	1 (2.8%)	0 (0%)	0.783
**Biological analysis**				
Hemoglobin (g/dL), IQR	15.1 (14.1-16.3)	15.0 (14.0-16.3)	15.4 (14.6-16.8)	0.776
Leukocyte count (10^3/µL), IQR	7.7 (5.9-9.7)	7,5 (5,9-9,6)	7,8 (5,8-9,8)	0.972
Platelet count (Giga/L), IQR	230 (199-275)	235 (171-269)	243 (205-266)	0.047
Partial thromboplastin time, PTT	98 (93-107)	97 (92-107)	103 (92-109)	0.649
Prothrombin time, PT (%), IQR	94 (84-107)	95 (87-110)	87 (74-106)	0.352
Fibrinogen (g/L), IQR	3.2 (2.9-3.8)	3.1 (2.9-3.5)	4.2 (3.2-7.0)	0.011
C-reactive protein (mg/dL), IQR	2.5 (0.6-7.2)	2.4 (0.5-5.2)	8.2 (0.6-8.0)	0.020
CPK (IU/L), IQR	229 (146-336)	223 (147-313)	238 (104-621)	0.026
ADAMTS13 activity (%), IQR	92.5 (77.5-116.8)	105 (88-129)	66 (62-74)	<0.001
**Treatment**				
Bothrofav® administration, n (%)	45 (98%)	35 (97%)	10 (10%)	0.783
Antibiotics, n (%)	12 (26%)	8 (22%)	4 (40%)	0.416

**Outcome**				
Ambulatory patients, n (%)	22 (48%)	18 (60%)	4 (40%)	0.725
Length of hospital stay (day), mean±SD	2.0±1.8	1.7±1.0	3.2±3.2	0.019
Deceased patients, n (%)	0 (0%)	0 (0%)	0 (0%)	na

Results are reported as median and inter-quartile range (IQR) or as absolutes number of cases and percentages. Abbreviation: ADAMTS13, a disintegrin-like and metalloproteinase with a thrombospondin type 1 motif, member 13; CPK, creatine phosphokinase; MRI, magnetic resonance imaging; na, non-applicable.

Multivariate linear regression was performed to test the relationship between length of hospital stay and independent predictors (Table 3).

<u>Table 3</u>: Analysis of the relationship between length of hospital stay and independent predictors (patient and envenoming characteristics): univariate and multivariate linear regression

	**Univariate analysis**			**Multivariate analysis**	
	**B-coefficient**	***β* coefficient**	**P value**	***β* coefficient**	**P value**
Age, years	0.032	0.278	0.061		
Delay for antivenom	<0.001	-0.068	0.698		
Grade severity of envenoming	1.399	0.456	0.001	0.486	0.002
Platelet count	0.005	-0.215	0.150		
Fibrinogen	0.704	0.444	0.010		
CPK	0.001	0.062	0.702		
C-reactive protein	0.015	0.415	0.004	0.291	0.054
ADAMTS13 activity	0.016	-0.259	0.082	-0.285	0.057

Abbreviation: ADAMTS13, a disintegrin-like and metalloproteinase with a thrombospondin type 1 motif, member 13; CPK, creatine phosphokinase. Beta coefficients (*β*-coefficient), i.e., standardized regression coefficients, are displayed to compare the relative strengths of our predictors. B-coefficient (B-coefficient) indicates the average increase in length of hospital stay associated with a 1-unit increase in a predictor.

Variables were considered for multivariate analysis after taking into account their clinical pertinence and significant association ($p<0.10$) in univariate analysis. Variables entered into the initial multivariate linear model for length of hospital stay: age, grade of the envenoming, fibrinogen, CPK, C-reactive protein and ADAMTS13 activity. A backward stepwise regression

approach was then applied to produce the final multivariate model, with a statistical significance level set at $p<0.05$. Multivariate linear regression retained only grade severity of envenoming as independent predictor of increase length of hospital stay, while reduced ADAMTS13 activity and increased C-reactive protein levels bordered on statistical significance.

DISCUSSION

A syndrome consistent with thrombotic microangiopathy may develop within the few days after snakebite envenoming and combines features such as thrombocytopenia, hemolytic anemia with presence of schistocytes in blood smears, decreased haptoglobin level, increased lactate dehydrogenase level and acute kidney injury [15,16]. While thrombotic microangiopathy has been occasionally described in *Bothrops* sp. envenoming [17-19,29-32], only few case reports have previously evaluated the levels of ADAMTS-13 [17,18,29-31], the main biological factor of endothelial dysfunction and thrombosis risk associated with this syndrome. For the first time, our study provided evidence suggesting that ADAMTS-13 activity is reduced in experimental venom exposure and in human *B. lanceolatus* envenoming. Our study results further suggest that, together with high grade severity score of envenoming and increased C-reactive protein, moderately reduced levels of ADAMTS-13 activity are associated with increased length of hospital stays.

ADAMTS13 is primarily synthesized in the liver, and its main function is to cleave von Willebrand factor (VWF) anchored on the endothelial surface, in circulation, and at the sites of vascular injury [20-23]–. The formation and elongation of VWF strings on endothelial surfaces are tightly regulated by ADAMTS13 through proteolytic cleavage of VWF strings. In the absence or reduction of ADAMTS13 activity, ultra-large von Willebrand factor strings remain anchored at the endothelium and can recruit flowing platelets and causing uncontrolled thrombosis in terminal arterioles and capillaries [20-23]. Along with endothelial inflammation and recruitment of immune competent cells, such deregulation of the ADAMTS13-vWF axis can play a critical role in the pathophysiology of thrombotic complications observed in human *B. lanceolatus* envenoming.

Our experimental results provide the new information that incubation of human plasma with known elevated ADAMTS13 activities with *B. lanceolatus* venom dose dependently reduced ADAMTS13 activity. In line, findings in *Bothrops* sp. envenoming have previously reported mild reduction of ADAMTS13 activity in rodents [26,27]. Such observations have been attributed to the proteolytic cleavage of ADAMTS13 by snake venom metalloproteinases (SVMP), because Na_2-EDTA (a SVMP inhibitor) may partially block these alterations [26]. It has been also shown that enzymatic activities of thrombin and plasmin, which typically generated in the systemic circulation during *Bothrops* sp. envenoming, can induced a proteolytic inactivation of ADAMTS13 [33]. The contrasting changes of ADAMTS13 activity displayed by *B. atrox* venom and *B. lanceolatus* venom are in line with their

opposite effects on hemostasis, as the former induced increased ADAMTS13 activity and hemorrhagic risk while the later induced reduced ADAMTS13 activity and risk of thrombotic events. Of note, mechanisms leading to reduced ADAMTS13 activity by *B. lanceolatus* venom and increased ADAMTS13 activity by *B. atrox* venom have to be elucidated. In addition to proteolytical inactivation [33], conformational changes of ADAMTS13, i.e., closed or open, have been consistently shown to alter its activity [34,35]. For example, disruption of the closed conformation of ADAMTS13 contributes to increased ADAMTS13 activity toward VWF [34].

Our clinical study suggests that some patients suffering from *B. lanceolatus* envenoming displayed moderately low ADAMTS13 activity. Compared with patients with normal ADAMTS13 activity, those with moderately low ADAMTS13 activity have no major changes in coagulation tests but displayed biological evidence of systemic inflammatory, such as higher fibrinogen and C-reactive protein levels. An increased risk of thrombosis is expected in patients with moderately low ADAMTS13 activity due to the imbalance of the ADAMTS13/VWF axis (decreased levels of ADAMTS13 and increased levels of VWF multimers), which exacerbates the thromboinflammatory response and in turn causes hypercoagulation, inhibition of fibrinolysis, neutrophil activation, and interaction with neutrophil extracellular traps [36-39]. Hence, it is proposed that reduced ADAMTS13 activity observed in some *B. lanceolatus* envenomed patients can be associated with an increased risk of thrombotic events.

New information provided by our study emphasizes prognosis factors that may independently and collectively pose a risk to *B. lanceolatus* envenoming patients [40,41]. Previously published risk factors of increased hospital stays and poor outcome in adults include delay from the bite to adequate antivenom therapy, acute renal failure and abnormal blood results such as thrombocytopenia, coagulation test derangements (prothrombin time > 13.2 s, partial thromboplastin time > 37.2 s, low fibrinogen levels), elevated creatinine kinase (CK), along with organ failure (respiratory failure, shock, neurotoxicity, ischemic stroke) associated with specific snake species [42-44]. Several scoring systems have been developed to risk-stratify patients with snakebite envenoming. The Snakebite Severity Score is an objective symptom severity scoring tool developed and validated for the evaluation of initial severity and crotaline envenomation progression and to guide antivenom therapy [43]. This score provides a comprehensive framework based on clinical assessment and coagulation tests to facilitate risk stratification of envenoming [42-44]. In our experience in Martinique, we used a simple scale to grade patients bitten by *B. lanceolatus*, which focuses on signs such as swelling, pain, and systemic manifestations [40, 41]. Minor presentations on this scale are grade 1, while severe swelling with or without systemic features is assigned to grade from 2 to 4 guide decision and dose for antivenom [6, 38,39]. In absence of mortality in our series, the present study confirms that the grading severity system used in Martinique was an independent predictor of length of hospital stay. Overall, multivariate linear regression retained only grade severity of envenoming as independent predictor of increase length of

hospital stay, while reduced ADAMTS13 activity and increased C-reactive protein levels bordered on statistical significance.

Limitations of the study

Several study limitations are to be noted in our exploratory research. Firstly, few reports have previously provided range of plasmatic venom concentration in *B. lanceolatus* envenoming. In our *in vitro* experimental studies, we have used venom concentration in the range of 10 to 1000 ng/mL, which is in line with those previous observations [38]. Of note, our series of *in vitro* experiments clearly suggests that reduced ADAMTS13 activity was a unique feature of *B. lanceolatus* venom because *B. atrox* venom induced opposite changes on ADAMTS13 activity. However, exact mechanisms that differentially alter ADAMTS13 activity in *B. lanceolatus* venom and *B. atrox* venom should be explored in much details.

Secondly, one significant concern pertains to the data collection process in this retrospective series of patients hospitalized at the University Hospital of Martinique. All data obtained in the present study was subject to review by the healthcare professionals of the snakebite group at the University Hospital of Martinique responsible for recording clinical information. Another main limitation is the moderate sample size of our study. Hence, the clinical consequences of reduced ADAMTS13 activity in *B. lanceolatus* envenoming was not studied in term of thrombosis. In this series of patients, specific outcome related to ischemic events, i.e., stroke, was not considered since only two cases of stroke were recorded. When considering length of hospital stay as an outcome, our results identified envenoming severity as an independent predictor, but reduced ADAMTS13 activity only bordered on statistical significance in the multivariate linear regression, possibly due to the small sample size of our study. In line with the reduced ADAMTS13 activity, determination of the level of plasmatic ultra-large von Willebrand multimer (ULVWF) was not available in this retrospective study. Indeed, increased ULVWF in the plasma as a consequence of would ascertain the prothrombotic role of reduced ADAMTS13 activity in *B. lanceolatus* envenoming. In our series, patients with reduced ADAMTS13 activity were older than those with normal activity. Hence, age (≥65) could have contributed to aberrant plasma indicators of endothelial coagulopathy such as ADAMTS13 [45]. Eventually, whether reduced ADAMTS13 activity is a specific feature of *B. lanceolatus* venom should be documented in humans by comparison with *Bothrops* sp. envenoming typically associated with consumption coagulopathy.

CONCLUSION

For the first time, our study provided evidence suggesting that ADAMTS-13 activity is reduced in experimental venom exposure and in human *B. lanceolatus* envenoming. Such moderately low ADAMTS13 activity may be implicated in the increased risk of thrombosis *B. lanceolatus* envenomed patients due to the imbalance of the ADAMTS13/VWF axis, which exacerbates biological features of thromboinflammation. Our study results further suggest that, together with high grade severity score of

envenoming and increased C-reactive protein, moderately reduced levels of ADAMTS-13 activity are associated with increased length of hospital stays.

STATEMENTS AND DECLARATIONS

Competing interests

The authors declare that they have no competing interests

Authors' contributions

All authors made substantial contributions as follows: 1) the conception and design of the study (RN, DR), 2) acquisition of data (JF, CR, O PL, FR, PJ, PF, HK), 3) analysis and interpretation of data (JF, CR, RN), 4) drafting the article (RN, DR), and 5) final approval of the version to be submitted (all authors).

Funding

The present study benefited from grants from

- APIDOM GIRCI SOHO, 2019
- French National Research Agency (ANR-25: AAPG 2025 - PRC – KariBothrops)

Ethics approval and consent to participate

Our manuscript includes a statement on ethics approval and consent and the name of the ethics committee that approved the study, as well as the committee’s reference number. All patients were managed in accordance with the amended Declaration of Helsinki (http://www.wma.net/en/30publications/10policies/b3/). The study was approved by the CHU Martinique hospital’s institutional review board (IRB #01072019).

The authors declare that generative AI and/or AI-assisted technologies have not been used in the writing process.

REFERENCES

1. Gutiérrez JM, Calvete JJ, Habib AG, Harrison RA, Williams DJ, Warrell DA. Snakebite envenoming. Nat Rev Dis Primers. 2017; 3:17079. doi: 10.1038/nrdp.2017.79.
2. Seifert SA, Armitage JO, Sanchez EE. Snake Envenomation. N Engl J Med. 2022; 386:68-78. doi: 10.1056/NEJMra2105228.
3. Monteiro WM, Contreras-Bernal JC, Bisneto PF, Sachett J, Mendonça da Silva I, Lacerda M, et al. *Bothrops atrox*, the most important snake involved in human envenomings in the amazon: How venomics contributes to the knowledge of snake biology and clinical toxinology. Toxicon X. 2020; 6:100037. doi: 10.1016/j.toxcx.2020.100037.
4. Resiere D, Kallel H, Florentin J, Houcke S, Mehdaoui H, Gutiérrez JM, Neviere R. *Bothrops* (Fer-de-lance) snakebites in the French departments of the Americas (Martinique and Guyana): Clinical and experimental studies and treatment by immunotherapy. PLoS Negl Trop Dis. 2023; 17:e0011083. doi: 10.1371/journal.pntd.0011083.
5. Resiere D, Hossein M, Megarbane B. Snake Bites by *Bothrops lanceolatus* in Martinique. Med Sante Trop. 2018; 28:37-43. doi: 10.1684/mst.2018.0760.
6. Resiere D, Mégarbane B, Valentino R, Mehdaoui H, Thomas L. *Bothrops lanceolatus* bites: guidelines for severity assessment and emergent management. Toxins (Basel). 2010; 2:163-73. doi: 10.3390/toxins2010163.
7. Thomas L, Chausson N, Uzan J, Kaidomar S, Vignes R, Plumelle Y, Bucher B, Smadja D. Thrombotic stroke following snake bites by the "Fer-de-Lance " *Bothrops lanceolatus* in Martinique despite antivenom treatment: a report of three recent cases. Toxicon. 2006; 48:23-8. doi: 10.1016/j.toxicon.2006.04.007.
8. Thomas L, Tyburn B, Lang J, Ketterle J. Early infusion of a purified monospecific F(ab')2 antivenom serum for *Bothrops lanceolatus* bites in Martinique. Lancet. 1996; 347(8998):406. doi: 10.1016/s0140-6736(96)90590-5.
9. Thomas L, Tyburn B, Bucher B, Pecout F, Ketterle J, Rieux D, Smadja D, Garnier D, Plumelle Y. Prevention of thromboses in human patients with *Bothrops lanceolatus* envenoming in Martinique: failure of anticoagulants and efficacy of a monospecific antivenom. Research Group on Snake Bites in Martinique. Am J Trop Med Hyg. 1995; 52:419-26. doi: 10.4269/ajtmh.1995.52.419.
10. Resiere D, Kallel H, Florentin J, Houcke S, Mehdaoui H, Gutiérrez JM, Neviere R. *Bothrops* (Fer-de-lance) snakebites in the French departments of the Americas (Martinique and Guyana): Clinical and experimental studies and treatment by immunotherapy. PLoS Negl Trop Dis. 2023; 17:e0011083. doi: 10.1371/journal.pntd.0011083.

11. Resiere D, Florentin J, Mehdaoui H, Kallel H, Legris-Allusson V, Gueye P, Neviere R. *Bothrops lanceolatus* Envenoming in Martinique: A Historical Perspective of the Clinical Effectiveness of Bothrofav Antivenom Treatment. Toxins (Basel). 2024; 16(3):146. doi: 10.3390/toxins16030146.

12. Rucavado A, Camacho E, Escalante T, Lomonte B, Fernández J, Solano D, Quirós-Gutiérrez I, Ramírez-Vargas G, Vargas K, Argüello I, Navarro A, Abarca C, Segura Á, Florentin J, Kallel H, Resiere D, Neviere R, Gutiérrez JM. A murine experimental model of the pulmonary thrombotic effect induced by the venom of the snake *Bothrops lanceolatus*. PLoS Negl Trop Dis. 2024; 18:e0012335. doi: 10.1371/journal.pntd.0012335.

13. Radouani F, Jalta P, Rapon C, Lezin C, Branford C, Florentin J, Gutierrez JM, Resiere D, Neviere R, Pierre-Louis O. The Contrasting Effects of *Bothrops lanceolatus* and *Bothrops atrox* Venom on Procoagulant Activity and Thrombus Stability under Blood Flow Conditions. Toxins (Basel). 2024; 16:400. doi: 10.3390/toxins16090400.

14. Larréché S, Chippaux JP, Chevillard L, Mathé S, Résière D, Siguret V, Mégarbane B. Bleeding and Thrombosis: Insights into Pathophysiology of *Bothrops* Venom-Related Hemostasis Disorders. Int J Mol Sci. 2021; 22:9643. doi: 10.3390/ijms22179643.

15. Noutsos T, Currie BJ, Wijewickrama ES, Isbister GK. Snakebite Associated Thrombotic Microangiopathy and Recommendations for Clinical Practice. Toxins (Basel). 2022; 14:57. doi: 10.3390/toxins14010057.

16. Noutsos T, Isbister GK. Snakebite-associated thrombotic microangiopathy: a spotlight on pharmaceutical interventions. Expert Rev Clin Pharmacol. 2023; 16:559-574. doi: 10.1080/17512433.2023.2220963.

17. Malaque CMS, Duayer IF, Santoro ML. Acute kidney injury induced by thrombotic microangiopathy in two cases of *Bothrops* envenomation. Clin Toxicol (Phila). 2019; 57:213-216. doi: 10.1080/15563650.2018.1510129.

18. Bucaretchi F, Pimenta MMB, Borrasca-Fernandes CF, Prado CC, Capitani EM, Hyslop S. Thrombotic microangiopathy following *Bothrops jararaca* snakebite: case report. Clin Toxicol (Phila). 2019; 57:294-299. doi: 10.1080/15563650.2018.1514621.

19. Mota SMB, Albuquerque PLMM, Silva Júnior GBD, Daher EF. Thrombotic microangiopathy due to *Bothrops erythromelas*: a case report in Northeast Brazil. Rev Inst Med Trop Sao Paulo. 2020; 62:e53. doi: 10.1590/s1678-9946202062053.

20. Hanna R, Gibney R, Boysen-Osborn M. Thrombotic Microangiopathies. Emerg Med Clin North Am. 2025; 43:407-420. doi: 10.1016/j.emc.2025.03.008.

21. Zhou Y, Moake J, Dong JF. Molecular basis of thrombotic microangiopathy. J Thromb Haemost. 2025; S1:538-7836(25)00400-3. doi: 10.1016/j.jtha.2025.06.014.

22. Azoulay E, Veyradier A, Cataland S, Joly BS, Knoebl P, Zafrani L, Wendon J, Fakhouri F, Lämmle B, Pavenski K, Biebuyck N, Vanhoorelbeke K, Chaturvedi S, Pishko AM, Donadello K, Scully M, Matsumoto M, Peyvandi F, Mingot-Castellano ME, Zheng XL, Mariotte E, Benhamou Y, Coppo P. Thrombotic thrombocytopenic purpura: early diagnosis and effective treatment in 2025. Intensive Care Med. 2025; 51:1225-1239. doi: 10.1007/s00134-025-07981-3.

23. Deshpande SR, Tarawneh H, Deitelzweig C, Tong J, Zhou T, Chen Y, Cuker A. Rapid ADAMTS13 activity assays for thrombotic thrombocytopenic purpura: a systematic review and meta-analysis. Blood. 2025; 146:233-246. doi: 10.1182/blood.2025028416.

24. Sonneveld MA, de Maat MP, Portegies ML, Kavousi M, Hofman A, Turecek PL, Rottensteiner H, Scheiflinger F, Koudstaal PJ, Ikram MA, Leebeek FW. Low ADAMTS13 activity is associated with an increased risk of ischemic stroke. Blood. 2015; 126:2739-46. doi: 10.1182/blood-2015-05-643338.

25. Sonneveld MA, Kavousi M, Ikram MA, Hofman A, Rueda Ochoa OL, Turecek PL, Franco OH, Leebeek FW, de Maat MP. Low ADAMTS-13 activity and the risk of coronary heart disease - a prospective cohort study: the Rotterdam Study. J Thromb Haemost. 2016; 14:2114-2120. doi: 10.1111/jth.13479.

26. Thomazini CM, Sachetto ATA, de Albuquerque CZ, de Moura Mattaraia VG, de Oliveira AK, Serrano SMT, Lebrun I, Barbaro KC, Santoro ML. Involvement of von Willebrand factor and botrocetin in the thrombocytopenia induced by *Bothrops jararaca* snake venom. PLoS Negl Trop Dis. 2021;15: e0009715. doi: 10.1371/journal.pntd.0009715.

27. Larréché S, Chevillard L, Jourdi G, Mathé S, Servonnet A, Joly BS, Siguret V, Chippaux JP, Mégarbane B. Bothrops venom-induced hemostasis disorders in the rat: Between Scylla and Charybdis. PLoS Negl Trop Dis. 2023; 17:e0011786. doi: 10.1371/journal.pntd.0011786.

28. Sachetto ATA, Jensen JR, Santoro ML. Liver gene regulation of hemostasis-related factors is altered by experimental snake envenomation in mice. PLoS Negl Trop Dis. 2020; 14:e0008379. doi: 10.1371/journal.pntd.0008379.

29. Ho WK, Verner E, Dauer R, Duggan J. ADAMTS-13 activity, microangiopathic haemolytic anaemia and thrombocytopenia following snake bite envenomation. Pathology. 2010; 42(2):200-2. doi: 10.3109/00313020903493955.

30. Casamento AJ, Isbister GK. Thrombotic microangiopathy in two tiger snake envenomations. Anaesth Intensive Care. 2011; 39:1124-7. doi: 10.1177/0310057X1103900622.

31. Fuchs J, Faber K, Tuchscherer DT, Tsakiris DA, Weiler S, Hofer KE. Bite by a juvenile *Bothrops venezuelensis* (Venezuelan lancehead) resulting in severe envenomation: A case report. Toxicon. 2020; 180:39-42. doi: 10.1016/j.toxicon.2020.04.002.

32. Malbranque S, Piercecchi-Marti MD, Thomas L, Barbey C, Courcier D, Bucher B, Ridarch A, Smadja D, Warrell DA. Fatal diffuse thrombotic microangiopathy after a bite by the "Fer-de-Lance" pit viper (*Bothrops lanceolatus*) of Martinique. Am J Trop Med Hyg. 2008; 78(6):856-61.

33. Crawley JT, Lam JK, Rance JB, Mollica LR, O'Donnell JS, Lane DA. Proteolytic inactivation of ADAMTS13 by thrombin and plasmin. Blood. 2005; 105:1085-93. doi: 10.1182/blood-2004-03-1101.

34. Bonnez Q, Vanhoorelbeke K. Open or closed? Understanding the molecular mechanisms and clinical implications of ADAMTS13's conformation. Hemasphere. 2025 Jul 27;9(7):e70189. doi: 10.1002/hem3.70189.

35. DeYoung V, Singh K, Kretz CA. Mechanisms of ADAMTS13 regulation. J Thromb Haemost. 2022; 20(12):2722-2732. doi: 10.1111/jth.15873.

36. Luka N, South K, Jones R, Unsworth AJ, Coutts G, Mosneag IE, Younas M, Bradley A, Wong SY, Collins E, Quigley C, Knight SB, McColl BW, McCulloch L, Grainger JR, Smith CJ, Allan SM. The Role of the VWF/ADAMTS13 Axis in the Thromboinflammatory Response in Ischemic Stroke After SARS-CoV2 Infection. Brain Behav. 2025; 15:e70348. doi: 10.1002/brb3.70348.

37. Kawecki C, Lenting PJ, Denis CV. von Willebrand factor and inflammation. J Thromb Haemost. 2017; 15(7):1285-1294. doi: 10.1111/jth.13696.

38. Peyvandi F, Garagiola I, Baronciani L. Role of von Willebrand factor in the haemostasis. Blood Transfus. 2011; Suppl 2:3-8. doi: 10.2450/2011.002S.

39. Gragnano F, Sperlongano S, Golia E, Natale F, Bianchi R, Crisci M, Fimiani F, Pariggiano I, Diana V, Carbone A, Cesaro A, Concilio C, Limongelli G, Russo M, Calabrò P. The Role of von Willebrand Factor in Vascular Inflammation: From Pathogenesis to Targeted Therapy. Mediators Inflamm. 2017; 2017:5620314. doi: 10.1155/2017/5620314.

40. Bucher B, Canonge D, Thomas L, Tyburn B, Robbe-Vincent A, Choumet V, Bon C, Ketterlé J, Lang J. Clinical indicators of envenoming and serum levels of venom antigens in patients bitten by *Bothrops lanceolatus* in Martinique. Research Group on Snake Bites in Martinique. Trans R Soc Trop Med Hyg. 1997; 91:186-90. doi: 10.1016/s0035-9203(97)90219-4.

41. Thomas L, Tyburn B, Ketterlé J, Biao T, Mehdaoui H, Moravie V, Rouvel C, Plumelle Y, Bucher B, Canonge D, Marie-Nelly CA, Lang J. Prognostic significance of clinical grading of patients envenomed by Bothrops lanceolatus in Martinique. Members of the Research Group on Snake Bite in Martinique. Trans R Soc Trop Med Hyg. 1998; 92:542-5. doi: 10.1016/s0035-9203(98)90907-5.

42. Wood D. Clinical Risk Factors Associated with Poor Outcomes in Snake Envenoming: A Narrative Review. Toxins (Basel). 2023; 15:675. doi: 10.3390/toxins15120675.

43. Dart RC, Hurlbut KM, Garcia R, Boren J. Validation of a severity score for the assessment of crotalid snakebite. Ann Emerg Med. 1996; 27:321-6. doi: 10.1016/s0196-0644(96)70267-6.

44. Fowler AL, Hughes DW, Muir MT, VanWert EM, Gamboa CD, Myers JG. Resource Utilization After Snakebite Severity Score Implementation into Treatment Algorithm of Crotaline Bite. J Emerg Med. 2017; 53:854-861. doi: 10.1016/j.jemermed.2017.08.095.

45. Thangaraju K, Katneni U, Akpan IJ, Tanaka K, Thomas T, Setua S, Reisz JA, Cendali F, Gamboni F, Nemkov T, Kahn S, Wei AZ, Valk JE, Hudson KE, Roh DJ, Moriconi C, Zimring JC, D'Alessandro A, Spitalnik SL, Francis RO, Buehler PW. The Impact of Age and BMI on the VWF/ADAMTS13 Axis and Simultaneous Thrombin and Plasmin Generation in Hospitalized COVID-19 Patients. Front Med (Lausanne). 2022; 8:817305. doi: 10.3389/fmed.2021.817305.

Data of the study

Click here to access/download
Supporting Information
46Pts_ADAM13_Bothrops.xlsx
