# Supplementary material for: ADAMTS13 in Bothrops lanceolatus snakebite envenoming: Crude venom-induced reduction of in vitro enzymatic activity and clinical correlation in snakebite patients
Source: PLoS Negl Trop Dis. 2025 Oct 29;19(10):e0013678. doi: 10.1371/journal.pntd.0013678 (PMC12591401; doi:10.1371/journal.pntd.0013678)
Supplement: Attachment — Submitted filename: plos neglected TD.docx [file pntd.0013678.s003.docx]

This study investigates the impact of *Bothrops lanceolatus* venom on ADAMTS-13 activity. The research includes both in vitro experiments and a clinical study of patients bitten by this snake. The findings suggest that *B. lanceolatus* venom reduces ADAMTS-13 activity in a dose-dependent manner, potentially increasing the risk of thrombosis in envenomed patients.

While the authors addressed a very interesting topic, I have some concerns that are detailed below:

**Title Accuracy:** The title “ADAMTS-13 activity is reduced by *Bothrops lanceolatus* snake venom: in vitro experiments and clinical correlation” does not reflect the use of *B. atrox* venom in the in vitro experiments. Please revise the title to accurately represent the study's scope or remove the in vitro experiments involving *B. atrox* venom.

**Objective:** In the main objectives, the study was designed to test whether plasmatic ADAMTS-13 activity would be reduced by in vitro exposure to *B. lanceolatus* venom (in contrast with *B. atrox* venom) and in humans bitten by *B. lanceolatus* snake. This objective refers to both in vitro and human experiments. However, the method section lacks detailed information about the in vitro experimental design. Please provide a comprehensive description of the in vitro methodology.

Also, please clearly detail the primary objective and any secondary objectives of this study.

**P6 – L163-164:** The sentence “Patients with the diagnosis of *B. lanceolatus* envenoming presenting at the University Hospital of Martinique from January 2019 to Janvier 2023 were retrospectively.” Is incomplete. Please correct

**In the method section**, authors said that they used crude venoms obtained from adult wild-caught *B. lanceolatus* (n=12) and *B. atrox* specimens 139 (n=5), captured in Martinique and French Guyana, respectively. Please provide further detail on the composition of these venoms.

**In the result section**, authors presented the “*In vitro effects of B. lanceolatus* venom on plasmatic ADAMTS13 activity”. While, the invitro study method was not clearly defined and the reported results refers to both *B. lanceolatus* and *B. atrox*.

Authors also suggest that reduced ADAMTS-13 activity was associated with more severe envenoming. However, this assertion is inconsistent with the data, as 70% of envenomed patients with ADAMTS-13 < 75% belong to Grade 2, and no patients in Grade 4 had ADAMTS-13 activity < 75%. Please reconcile this discrepancy.

Authors performed a multivariate linear regression to test independent predictors of length of hospital stay. Please explain the rationale for conducting this comparison, as it does not appear to align with the stated objectives of the study. It feels out of the scope and doesn't significantly enhance the results.

Same remark on the discussion section where authors discuss factors associated to unfavorable outcome (P14 – L 315 to P15 – L 334). This is not in the objective of the study and doesn't contribute substantially to the reader's understanding of the primary findings.

**In the result section**, table 1 and 2, authors give patients characteristics but omit details on biological renal function, haptoglobin, schistocytes, LDH, and factor VIII level. These parameters are essential to interpret the data presented in this study and explain the precise mechanism of the envenoming.

**Figure 1:** ADAMTS-13 activity in serum exposed to 1000 ng/ml *B. atrox* venom was lower than in serum exposed to 10 and 100 ng/ml. Please check this result for accuracy and give explanation. Furthermore, given that the primary focus of this study is the effect of *B. lanceolatus* venom on ADAMTS-13 activity, the concurrent investigation of *B. atrox* venom is potentially confusing. These two venoms exhibit different effects on coagulation and induce distinct clinical manifestations, which could dilute the clarity of the core findings.

**Table 1:** the variable “ADAMTS13 activity <75%” is redundant since Q1 for ADAMTS13 activity was 77.5%

While reduced ADAMTS-13 activity is linked to increased thrombosis risk, the study doesn't directly demonstrate a higher incidence of thrombotic events in patients with reduced ADAMTS-13. Please explain this limitation in detail.

Could authors elaborate on the potential mechanisms by which *B. lanceolatus* venom reduces ADAMTS-13 activity?

**P6 - L164 and Page 8 - L 217:** please change “janvier” to “January”

**In the whole manuscript:** Authors used Adamst13, ADAMTS13, and ADAMTS-13. Please use ADAMTS-13 for uniformity.

Please discuss the limitations of the study more explicitly.
