# Supplementary material for: ADAMTS13 in Bothrops lanceolatus snakebite envenoming: Crude venom-induced reduction of in vitro enzymatic activity and clinical correlation in snakebite patients
Source: PLoS Negl Trop Dis. 2025 Oct 29;19(10):e0013678. doi: 10.1371/journal.pntd.0013678 (PMC12591401; doi:10.1371/journal.pntd.0013678)
Supplement: Attachment — Submitted filename: Comments.docx [file pntd.0013678.s005.docx]

**Dear Editor,**

Thank you for the opportunity tp review this manuscript. The study investigates a very interesting and important clinical topic. The paper is well-written and well-structured.

However, I have some major concerns that need to be addressed before the manuscript can be considered for publication, as following:

**References**

I noted several references in the bibliography that lack a corresponding in-text citation. For example:

2. Seifert SA, Armitage JO, Sanchez EE. Snake Envenomation. N Engl J Med. 2022; 386:68-78. doi: 10.1056/NEJMra2105228.

5. Resiere D, Hossein M, Megarbane B. Snake Bites by Bothrops lanceolatus in Martinique. Med 407 Sante Trop. 2018; 28:37-43. doi: 10.1684/mst.2018.0760.

8. Thomas L, Tyburn B, Lang J, Ketterle J. Early infusion of a purified monospecific F(ab')2antivenom serum for Bothrops lanceolatus bites in Martinique. Lancet. 1996; 347(8998):406. doi:

10.1016/s0140-6736(96)90590-5.

9. Thomas L, Tyburn B, Bucher B, Pecout F, Ketterle J, Rieux D, Smadja D, Garnier D, PlumelleY. Prevention of thromboses in human patients with Bothrops lanceolatus envenoming in

Martinique: failure of anticoagulants and efficacy of a monospecific antivenom. Research Group

on Snake Bites in Martinique. Am J Trop Med Hyg. 1995; 52:419-26. doi:10.4269/ajtmh.1995.52.419.

13. Radouani F, Jalta P, Rapon C, Lezin C, Branford C, Florentin J, Gutierrez JM, Resiere D, Neviere R, Pierre-Louis O. The Contrasting Effects of *Bothrops lanceolatus* and *Bothrops atrox* Venom

on Procoagulant Activity and Thrombus Stability under Blood Flow Conditions. Toxins (Basel).2024; 16:400. doi: 10.3390/toxins16090400.

Please conduct a complete check of the reference list against the text. All references must be cited, and any uncited references should be removed.

I have a major concern about the lines {**357, 358, 359**} where author says (**In our series, patients with reduced ADAMTS13 activity were older than those with normal activity. Hence, age ≥65 could have contributed to aberrant plasma indicators of endothelial coagulopathy such as ADAMTS13**) which I believe it is not a limitation, instead its one of the major parameter. Since, older age can change blood measurement like ADAMTS13, fibrinogen, and CRP by itself. Could the author do a new statistical test to see if the difference in fibrinogen is because of the snake venom, or just because that group was older.

To the discussion part, please add a sentence about the normal levels of these biological markers for a healthy people over 65 years old.

Greetings,
