# Supplementary material for: ADAMTS13 in Bothrops lanceolatus snakebite envenoming: Crude venom-induced reduction of in vitro enzymatic activity and clinical correlation in snakebite patients
Source: PLoS Negl Trop Dis. 2025 Oct 29;19(10):e0013678. doi: 10.1371/journal.pntd.0013678 (PMC12591401; doi:10.1371/journal.pntd.0013678)
Supplement: Attachment — Submitted filename: Reply to Reviewers Plos.docx [file pntd.0013678.s007.docx]

**Reply to Reviewers Plos_NTD-D-25-01531**

**Reviewers' Comments:**

Reviewer's Responses to Questions

**Key Review Criteria Required for Acceptance?**
As you describe the new analyses required for acceptance, please consider the following:
**Methods:**
-Are the objectives of the study clearly articulated with a clear testable hypothesis stated?
-Is the study design appropriate to address the stated objectives?
-Is the population clearly described and appropriate for the hypothesis being tested?
-Is the sample size sufficient to ensure adequate power to address the hypothesis being tested?
-Were correct statistical analysis used to support conclusions?
-Are there concerns about ethical or regulatory requirements being met?

Reviewer #1: YES

Reviewer #2: (No Response)

**Results:**
-Does the analysis presented match the analysis plan?
-Are the results clearly and completely presented?
-Are the figures (Tables, Images) of sufficient quality for clarity?

Reviewer #1: YES

Reviewer #2: (No Response)

**Conclusions:**

-Are the conclusions supported by the data presented?
-Are the limitations of analysis clearly described?
-Do the authors discuss how these data can be helpful to advance our understanding of the topic under study?
-Is public health relevance addressed?

Reviewer #1: YES

Reviewer #2: (No Response)

**Editorial and Data Presentation Modifications?**
Use this section for editorial suggestions as well as relatively minor modifications of existing data that would enhance clarity. If the only modifications needed are minor and/or editorial, you may wish to recommend “Minor Revision” or “Accept”.

Reviewer #1: MAJOR REVISION

Reviewer #2: (No Response)

**Summary and General Comments:**

Use this section to provide overall comments, discuss strengths/weaknesses of the study, novelty, significance, general execution and scholarship. You may also include additional comments for the author, including concerns about dual publication, research ethics, or publication ethics. If requesting major revision, please articulate the new experiments that are needed.

**Reviewer #1**:

This study investigates the impact of *Bothrops lanceolatus* venom on ADAMTS13 activity. The research includes both in vitro experiments and a clinical study of patients bitten by this snake. The findings suggest that *B. lanceolatus* venom reduces ADAMTS13 activity in a dose-dependent manner, potentially increasing the risk of thrombosis in envenomed patients. While the authors addressed a very interesting topic, I have some concerns that are detailed below:

**Title Accuracy:** The title “ADAMTS13 activity is reduced by *Bothrops lanceolatus* snake venom: in vitro experiments and clinical correlation” does not reflect the use of *B. atrox* venom in the in vitro experiments. Please revise the title to accurately represent the study's scope or remove the in vitro experiments involving *B. atrox* venom.

**Authors**: Indeed, the scope of our study involved *B. lanceolatus* snake. Please find the following title: ***ADAMTS13 in Bothrops lanceolatus snakebite envenoming: crude venom-induced reduction of in vitro enzymatic activity and clinical correlation in snakebite patients.*** Please understand that the data related to *Bothrops atrox* snake have been removed throughout the revised manuscript.

**Objective:** In the main objectives, the study was designed to test whether plasmatic ADAMTS13 activity would be reduced by in vitro exposure to *B. lanceolatus* venom (in contrast with *B. atrox* and in humans bitten by *B. lanceolatus* snake. This objective refers to both in vitro and human experiments. However, the method section lacks detailed information about the in vitro experimental design. Please provide a comprehensive description of the in vitro methodology.

Also, please clearly detail the primary objective and any secondary objectives of this study.

**Authors**: We fully agree with reviewer’s comment. Information regarding the *in vitro* experimental design was not accurately described. This specific issue has been considered in the revised Methods section of the manuscript. Please see page 6-7 line 155-165 for modifications in the revised (marked version) manuscript.

**P6 – L163-164:** The sentence “Patients with the diagnosis of *B. lanceolatus* envenoming presenting at the University Hospital of Martinique from January 2019 to Janvier 2023 were retrospectively.” Is incomplete. Please correct

**Authors**: We apologize for this confusing wording. Please read (page 7 line 182-183) the following in the revised (marked version) manuscript “*Patients with the diagnosis of B. lanceolatus envenoming presenting at the University Hospital of Martinique from January 2019 to Janvier 2023 were retrospectively include in the study*.”

**In the method section**, authors said that they used crude venoms obtained from adult wild-caught *B. lanceolatus* (n=12) and *B. atrox* specimens (n=5), captured in Martinique and French Guyana, respectively. Please provide further detail on the composition of these venoms.

**Authors**: As required, we have provided information on snakes captured in Martinique. Please read (page 6 line 142-146) in the revised (marked version) manuscript: “*Crude venom was obtained from 6 adult females and 6 males (112 to 156 cm long) caught in the wild in various locations of Martinique. Median (25th-75th percentiles) length of snakes was 168 (150-172) cm in female specimens and 158 (123-166) cm in male specimens.”.*

**In the result section**, authors presented the “*In vitro effects of B. lanceolatus* venom on plasmatic ADAMTS13 activity”. While, the *in vitro* study method was not clearly defined and the reported results refers to both *B. lanceolatus* and *B. atrox*.

**Authors**: As required, we have provided information regarding the *in vitro* ADAMTS13 experimental protocol for *B. lanceolatus* venom in the revised (marked version) manuscript (page 6-7 line 155-165). Please understand that *in vitro* ADAMTS13 activity experiments were performed on plasmatic samples prepared from commercialized control human plasma. Please read the following subtitle “*In vitro effects of B. lanceolatus venom on plasmatic ADAMTS13 activity in plasmatic samples prepared from commercialized control human plasma*” (page 8 line 221-222) in the revised (marked version) manuscript).

**In the result section**, Authors also suggest that reduced ADAMTS13 activity was associated with more severe envenoming. However, this assertion is inconsistent with the data, as 70% of envenomed patients with ADAMTS13 < 75% belong to Grade 2, and no patients in Grade 4 had ADAMTS13 activity < 75%. Please reconcile this discrepancy.

**Authors**: According to analysis of our results displayed Table 2 (line entitled “Grade severity of envenoming”), it was considered that reduced ADAMTS13 activity <75% (n=10 patients) was not associated with more severe envenoming (page 16 line 341-344 of the revised (marked version) manuscript). However, it was stated that “*This group of 10 patients (with reduced ADAMTS13 activity <75%) were older and displayed a lower platelet count (which remain normal), a more intense myonecrosis along with higher fibrinogen and C-reactive protein levels (Table 2) compared with patients with normal ADAMTS13 activity*.”

We agree that the place of the paragraph related the multivariate linear regression (Table 3) immediately after the Table 2 can induce some confusion in the reading. However, we confirm that the multivariate linear regression was not designed to test whether low ADAMTS13 activity was an independent predictor of snake bite severity (severity grade). Instead, this analysis aimed to test for independent predictors of hospital stay, which identified snake bite severity.

Authors performed a multivariate linear regression to test independent predictors of length of hospital stay. Please explain the rationale for conducting this comparison, as it does not appear to align with the stated objectives of the study. It feels out of the scope and doesn't significantly enhance the results. Same remark on the discussion section where authors discuss factors associated to unfavorable outcome (P14 – L 315 to P15 – L 334). This is not in the objective of the study and doesn't contribute substantially to the reader's understanding of the primary findings.

**Authors**: We fully agree with the reviewer’ comment that conducting analyzes aimed to test independent predictors of length of hospital stay can appear out of the scope and may not align with the objectives of the study. In our study, the number of thrombotic events (which may be mechanistically related to low ADAMTS13 levels) was quite low. This small sample size (n=2) has precluded any consistent analysis aimed to identify low ADAMTS13 levels as an independent predictor of thrombotic events in patients suffering *B. lanceolatus* envenoming. However, while our results gave interesting insight into ADAMTS13 levels in snakebite envenoming, clinical relevancy and prognostic association of low ADAMTS13 levels remain uncertain.

Because low ADAMTS13 levels has been consistently associated with end-organ failure, longer hospital stays and increased mortality in various pathologies (see references 24,25,46-48 on sepsis, thrombotic thrombocytopenic purpura, COVID, cardiovascular ischemia), we investigated as a secondary objective whether low ADAMTS13 level (among other) was an independent predictor increased length of hospital stay in patients suffering *B. lanceolatus* envenoming. Overall, the rationale for the multivariate linear regression to test independent predictors of length of hospital stay has been implemented with statements in the objectives of study (page 6 line 137 138), results section (page 14 line 176-180) and discussion part of the revised (marked version) manuscript.

**In the result section**, table 1 and 2, authors give patients characteristics but omit details on biological renal function, haptoglobin, schistocytes, LDH, and factor VIII level. These parameters are essential to interpret the data presented in this study and explain the precise mechanism of the envenoming.

**Authors**: We have understood that the reviewer’ comment addressed the question of microangiopathy and hemolytic anemia induced by snake venoms. In our experience in Martinique, this specific complication is not observed in the case of *Bothrops lanceolatus* envenoming. Hence, the biological documentation of hemolysis is not routinely performed in patients bitten by *Bothrops lanceolatus* snake hospitalized at the University Hospital of Martinique. In line with the reviewer’ comment, this specific issue has added to the limitations of the study part of the revised (marked version) manuscript (page 17 line 392-397).

**Figure 1:** ADAMTS13 activity in serum exposed to 1000 ng/ml *B. atrox* venom was lower than in serum exposed to 10 and 100 ng/ml. Please check this result for accuracy and give explanation. Furthermore, given that the primary focus of this study is the effect of *B. lanceolatus* venom on ADAMTS13 activity, the concurrent investigation of *B. atrox* venom is potentially confusing. These two venoms exhibit different effects on coagulation and induce distinct clinical manifestations, which could dilute the clarity of the core findings.

**Authors**: As required by the reviewer in the “Title Accuracy” comment, we have omitted the results of ADAMTS13 activity in plasma exposed to *B. atrox* venom. We agree that the concurrent investigation of *B. atrox* venom is potentially confusing.

**Table 1:** the variable “ADAMTS13 activity <75%” is redundant since Q1 for ADAMTS13 activity was 77.5%.

**Authors**: The line entitled “ADAMTS13 activity <75%” has been removed Table 1 of the revised manuscript.

While reduced ADAMTS13 activity is linked to increased thrombosis risk, the study doesn't directly demonstrate a higher incidence of thrombotic events in patients with reduced ADAMTS13. Please explain this limitation in detail.

**Authors**: As recommended by the reviewer, we have identified this specific issue as one of the major limitations of our study. Indeed, thrombotic events are the major complications seen in *B. lanceolatus* envenoming, but the small sample size of our study precluded definite conclusion implicating reduced ADAMTS13 level in the mechanisms of these thrombotic events. This issue is stated in the “Study limits” part of the revised manuscript.

Could authors elaborate on the potential mechanisms by which *B. lanceolatus* venom reduces ADAMTS13 activity?

**Authors**: As recommended by the reviewer’ comments, potential mechanisms for why B. lanceolatus venom reduces ADAMTS13 activity have been extensively discussed in the revised (marked version) manuscript (see page 15 line 318-337).

**P6 - L164 and Page 8 - L 217:** please change “janvier” to “January”

**Authors**: We apologize for these confusing wordings. Please read the correct “January” wording in the revised manuscript.

**In the whole manuscript:** Authors used Adamst13, ADAMTS13, and ADAMTS13. Please use ADAMTS13 for uniformity.

**Authors**: We apologize for these confusing wordings. ADAMTS13 has been uniformly used in the revised (marked version) manuscript. The word “Adamst13” is used for gene expression (see page 6 line 130).

**In the limitation of the study**: Please discuss the limitations of the study more explicitly.

**Authors**: In line with the reviewer’ comments we have discussed the limitation of the study more explicitly. Precisely, we have extended the limitation discussion on the reviewer’ comment who stated that “*while reduced ADAMTS13 activity is linked to increased thrombosis risk, the study doesn't directly demonstrate a higher incidence of thrombotic events in patients with reduced ADAMTS13*”.

**Reviewer #2**: (No Response)

**Dear Editor,**

Thank you for the opportunity to review this manuscript. The study investigates a very interesting and important clinical topic. The paper is well-written and well-structured.

**Authors**: We thank the reviewer for this comment.

However, I have some major concerns that need to be addressed before the manuscript can be considered for publication, as following: **References**

I noted several references in the bibliography that lack a corresponding in-text citation. For example:

2. Seifert SA, Armitage JO, Sanchez EE. Snake Envenomation. N Engl J Med. 2022; 386:68-78. doi: 10.1056/NEJMra2105228.

5. Resiere D, Hossein M, Megarbane B. Snake Bites by Bothrops lanceolatus in Martinique. Med 407 Sante Trop. 2018; 28:37-43. doi: 10.1684/mst.2018.0760.

8. Thomas L, Tyburn B, Lang J, Ketterle J. Early infusion of a purified monospecific F(ab')2antivenom serum for Bothrops lanceolatus bites in Martinique. Lancet. 1996; 347(8998):406. doi: 10.1016/s0140-6736(96)90590-5.

9. Thomas L, Tyburn B, Bucher B, Pecout F, Ketterle J, Rieux D, Smadja D, Garnier D, PlumelleY. Prevention of thromboses in human patients with Bothrops lanceolatus envenoming in Martinique: failure of anticoagulants and efficacy of a monospecific antivenom. Research Group on Snake Bites in Martinique. Am J Trop Med Hyg. 1995; 52:419-26. doi:10.4269/ajtmh.1995.52.419.

13. Radouani F, Jalta P, Rapon C, Lezin C, Branford C, Florentin J, Gutierrez JM, Resiere D, Neviere R, Pierre-Louis O. The Contrasting Effects of *Bothrops lanceolatus* and *Bothrops atrox* Venom on Procoagulant Activity and Thrombus Stability under Blood Flow Conditions. Toxins (Basel).2024; 16:400. doi: 10.3390/toxins16090400.

Please conduct a complete check of the reference list against the text. All references must be cited, and any uncited references should be removed.

**Authors**: We have double-checked the reference list as recommended by the reviewer. All references were cited in the previous manuscript. Please note that reference #2 (Seifert et al.) was cited line 87 of the initial manuscript as “[1-3]”, reference #5 (Resiere et al.) was cited line 89 of the initial manuscript as “[4-6]”, reference #8 and reference #9 (Thomas et al, 1996, 1995) were cited line 89 of the initial manuscript as “[7-11]”, reference #13 (Radouani et al.) was cited line 100 of the initial manuscript as “[10,12-14]”.

I have a major concern about the lines {**357, 358, 359**} where author says (**In our series, patients with reduced ADAMTS13 activity were older than those with normal activity. Hence, age ≥65 could have contributed to aberrant plasma indicators of endothelial coagulopathy such as ADAMTS13**) which I believe it is not a limitation, instead its one of the major parameter. Since, older age can change blood measurement like ADAMTS13, fibrinogen, and CRP by itself. Could the author do a new statistical test to see if the difference in fibrinogen is because of the snake venom, or just because that group was older. To the discussion part, please add a sentence about the normal levels of these biological markers for a healthy people over 65 years old.

**Authors**: We thank the reviewer for these valuable comments. Indeed, advanced age is one of the main factors affecting the levels of many biological parameters. According to reviewer’ comment we have removed the cited sentence of the limitation paragraph of the revised manuscript. According to reviewer’ comment, we have run a new set of statistical analysis to test whether levels of main biological parameters (ADAMTS13, fibrinogen, CRP, etc etc) were different in older patients (>65year old) compared by younger patients. No statistical difference was found just because the group was older. Please find the results of these statistical analysis in the supplemental Table S1 of the revised manuscript. This new information has been mentioned in the discussion part of the revised (marked version) manuscript (page 17 line 372-378).
